# Interlayer Friction and Adhesion Effects in Penta‐PdSe_2_‐Based van der Waals Heterostructures

**DOI:** 10.1002/advs.202400395

**Published:** 2024-07-04

**Authors:** Guoliang Ru, Weihong Qi, Shu Sun, Kewei Tang, Chengfeng Du, Weimin Liu

**Affiliations:** ^1^ State Key Laboratory of Solidification Processing and Center of Advanced Lubrication and Seal Materials Northwestern Polytechnical University Xi'an 710072 China; ^2^ Shandong Laboratory of Yantai Advanced Materials and Green Manufacturing Yantai 265503 China; ^3^ State Key Laboratory of Solid Lubrication Lanzhou Institute of Chemical Physics Chinese Academy of Sciences Lanzhou 730000 China

**Keywords:** 2D heterostructure, interlayer friction, superlubricity, van der Waals interaction

## Abstract

Due to their inherent lattice mismatch characteristics, 2D heterostructure interfaces are considered ideal for achieving stable and sustained ultralow friction (superlubricity). Despite extensive research, the current understanding of how interface adhesion affects interlayer friction remains limited. This study focused on graphene/MoS_2_ and graphene/PdSe_2_ heterostructure interfaces, where extremely low friction coefficients of ≈10^−3^ are observed. In contrast, the MoS_2_/PdSe_2_ heterostructure interfaces exhibit higher friction coefficients, ≈0.02, primarily due to significant interfacial interactions driven by interlayer charge transfer, which is closely related to the ionic nature of 2D material crystals. These findings indicate that the greater the difference in ionicity between the two 2D materials comprising the sliding interfaces is, the lower the interlayer friction, providing key criteria for designing ultralow friction pairs. Moreover, the experimental results demonstrate that interlayer friction in heterostructure systems is closely associated with the material thickness and interface adhesion strength. These experimental findings are supported by molecular dynamics simulations, further validating the observed friction behavior. By integrating experimental observations with simulation analyses, this study reveals the pivotal role of interface adhesion in regulating interlayer friction and offers new insights into understanding and optimizing the frictional performance of layered solid lubricants.

## Graphical abstract

The greater the difference in ionicity between the two 2D materials comprising the sliding interfaces is, the lower the interlayer friction, providing key criteria for designing ultralow friction 2D material friction pairs.

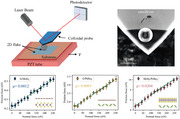

## 1 Introduction

The investigation of interface friction at the microscale has revealed numerous complex phenomena that determine the interactions between material surfaces. Adhesion and friction play a pivotal role in mechanical systems, as they directly influence energy dissipation^[^ ^1^ ^]^ and wear at the interface.^[^ ^1^, ^2^ ^]^ A profound understanding and control of friction and adhesion at different contact interfaces can lead to significant advancements in reducing energy losses, extending the lifespan of mechanical systems, and promoting innovation in new technologies. 2D materials have become a focal point of research in a wide range of materials due to their exceptional mechanical strength,^[^ ^3^ ^]^ electrical conductivity,^[^ ^4^ ^]^ and thermal stability,^[^ ^5^ ^]^ stemming from their atomic to few‐atom thickness. A significant advantage lies in the ease with which various 2D materials can be stacked, surpassing the combination possibilities of any traditional growth methods. Significant research efforts have been devoted to investigating the physical and chemical properties, as well as the synthesis and characterization, of 2D materials, including graphene and transition metal dichalcogenides.^[^ ^6^ ^]^ The ultrathin nature of 2D materials results in a high surface area‐to‐volume ratio, significantly influencing their friction and adhesion behavior with other materials. The understanding of interlayer adhesion and friction behavior in 2D materials is of paramount importance not only for fundamental scientific understanding but also for its significant implications in various applications. These applications encompass micro/nanoelectromechanical systems (MEMS/NEMS) and extend to a broad range of fields.^[^ ^7^ ^]^

Superlubricity is a highly sought‐after state in the field of tribology and is characterized by extremely low friction. Achieving superlubricity in 2D materials can significantly reduce energy losses caused by friction, potentially leading to revolutionary changes in various technological applications. Dienwiebel et al. first experimentally demonstrated structural superlubricity.^[^ ^8^ ^]^ They confirmed the lattice mismatch between two sliding graphite sheets through experimental measurements of the rotation effect of a graphite thin film, leading to ultralow friction behavior. In 2012, achieving microscale superlubricity under ambient pressure conditions marked a crucial milestone.^[^ ^9^ ^]^ Subsequent advancements have extended to centimeter‐long double‐walled carbon nanotubes,^[^ ^10^ ^]^ vdW heterostructures, such as MoS_2_/MoSe_2_,^[^ ^11^ ^]^ G/h‐BN,^[^ ^12^ ^]^ and macroscopic multicontact interfaces,^[^ ^13^ ^]^ among others. Due to the relatively low out‐of‐plane bending stiffness of graphene, it easily protrudes (wrinkles) ahead of the sliding atomic force microscopy (AFM) tip, and this friction phenomenon exhibits a distinct layer thickness effect.^[^ ^14^ ^]^ This phenomenon is notably observed on silicon dioxide and copper substrates and is governed by the adhesion force between 2D graphene materials and the substrate. Zeng et al. conducted a comprehensive comparative study on the dynamic friction and adhesion properties of various graphene materials, including pristine graphene, graphene oxide, and fluorinated graphene, using lateral force microscopy (LFM).^[^ ^15^ ^]^ The experiments revealed strong adhesion and friction enhancement in graphene oxide. Interestingly, in certain cases, low friction corresponded to high adhesion forces. Mica surfaces in humid air exhibit very low friction, but due to capillary action, the adhesion energy is exceptionally high.^[^ ^16^ ^]^ This behavior indicates a much more complex relationship between adhesion and friction forces, making it a crucial frontier in the study of tribology.

The relationship between the performance and structure of 2D materials is of utmost importance, as changes in crystal structure can greatly alter a material's properties. In recent years, Wang and his colleagues introduced a novel meta‐stable carbon allotrope called pentagon graphene (PG), which is composed entirely of irregular pentagons arranged similarly to a tiled pentagon pattern.^[^ ^17^ ^]^ Recent reports indicate that van der Waals (vdW) heterostructures composed of pentagon graphene exhibit robust superlubricity.^[^ ^18^ ^]^ PdSe_2_ is the first experimentally confirmed material with a pentagonal 2D structure, differing from common (1T or 2H) hexagonal crystal structures. Its unique folded pentagonal structure gives it an unusual negative Poisson's ratio^[^ ^19^ ^]^ and in‐plane optical^[^ ^20^ ^]^ and electrical properties.^[^ ^21^, ^22^ ^]^ In this study, it was unexpectedly found that heterostructure interfaces composed of PdSe_2_ (natural incommensurate contact interfaces) also exhibit high frictional resistance when aligned. Generally, the adhesion and friction forces in layered materials are not yet fully understood. Therefore, it is essential to further determine the influence of interfacial adhesion on interlayer friction.

Numerous experimental investigations in the field of nanotribology have utilized AFM, which enables single‐asperity contact with the substrate. In this research paper, a study was conducted on the interlayer friction of 2D materials employing custom‐made adhesive colloidal probes coated with 2D monolayers. Remarkably, a correlation was uncovered between the interlayer friction behavior of these 2D materials and their ionicity (polarizability). This discovery provides intriguing insight into the relationship between material properties and friction characteristics at the nanoscale. Considering the dipole‒dipole interactions in MoS_2_/PdSe_2_, polarization occurs at the interface (high interlayer charge transfer), resulting in remarkably high friction coefficients in the MoS_2_/PdSe_2_ heterostructure system. Conversely, the friction coefficients on the surfaces of the G/MoS_2_ and G/PdSe_2_ systems are on the order of 10^−3^, indicating a state of superlubricity. Furthermore, this phenomenon was analyzed and validated at the atomic scale using molecular dynamics (MD) simulations.

## 2 Results

### 2.1 Gold‐Assisted Mechanical Exfoliation of Large‐Area 2D PdSe_2_

**Figure 1:**
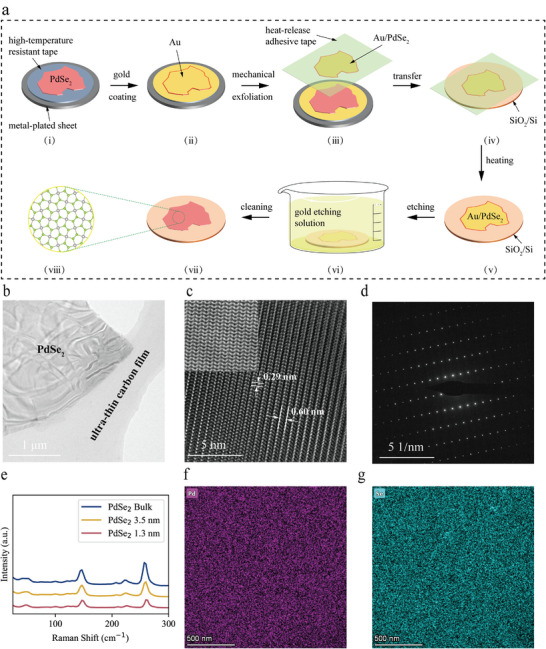
Gold‐assisted exfoliation and characterization of large‐scale 2D PdSe_2_ materials. a) Schematic representation of gold‐assisted mechanical exfoliation utilized to obtain large‐area monolayer and few‐layer PdSe_2_ materials. b) Bright‐field image showcasing few‐layer 2D PdSe_2_ on a porous carbon microgrid. c) High‐resolution transmission electron microscopy (HRTEM) image of few‐layer 2D PdSe_2_, with an inset depicting an atomic‐resolution STEM image in the upper left corner. d) Fast Fourier transform (FFT) image of few‐layer 2D PdSe_2_. e) Raman spectroscopy of 2D PdSe_2_ materials with varying thicknesses. f,g) Imagery from energy dispersive spectroscopy (EDS) reveals the spatial arrangement of Pd and Se within few‐layer 2D PdSe_2_.

The methods for preparing atomically thin 2D nanosheets are diverse and include mechanical exfoliation,^[^ ^23^ ^]^ chemical exfoliation,^[^ ^24^ ^]^ chemical vapor deposition,^[^ ^25^ ^]^ and liquid exfoliation.^[^ ^26^ ^]^ Despite its limited scalability, mechanical exfoliation remains the most effective method for producing the cleanest, most highly crystalline, and atomically thin 2D layered material nanosheets. The preparation of large‐area and pristine 2D materials constitutes an initial pivotal step. Traditional tape‐based mechanical exfoliation techniques have proven effective for the procurement of monolayer and few‐layer graphene and MoS_2_.^[^ ^27^ ^]^ However, such methods are ill suited for the PdSe_2_ system, often resulting in exfoliated PdSe_2_ flakes exceeding 40 nm in thickness, thereby thwarting the attainment of few‐layer PdSe_2_. Through a blend of theoretical and empirical scrutiny, Huang et al. elucidated that gold surfaces can furnish substantial attractive forces to surmount the interlayer interactions inherent in 2D materials terminated with chalcogen (VIA) and halogen (VIIA) elements, with minimal perturbation to the intrinsic electronic structural attributes of the materials, thereby earmarking them as ideal interfaces to facilitate 2D material exfoliation.^[^ ^28^ ^]^ Herein, gold‐assisted exfoliation of 2D PdSe_2_ materials was implemented, as depicted in **Figure** **1**a.

Initially, block specimens of PdSe_2_ were placed on a metallic coated plate furnished with high‐temperature‐resistant tape and gently pressed to ensure optimal contact. Subsequently, a vacuum coating apparatus was used to deposit a thin layer of Au (≈100 nm) onto the substrate bearing the specimens. By utilizing thermal release tape adhered to the gold film surface, slow peeling facilitated the exfoliation of monolayer or few‐layer 2D PdSe_2_ from the topmost layer of the PdSe_2_ specimen, which was then positioned on the target substrate and heated for 5 min to accomplish specimen transfer. Ultimately, a gold etching solution was applied to etch the gold film on the specimen surface, yielding multiple large‐area monolayer or few‐layer PdSe_2_ flakes. A comprehensive elucidation of the preparation procedure is provided in Note 1 (Supporting Information).

High‐angle annular dark field scanning transmission electron microscopy (HAADF‐STEM) was utilized to elucidate the atomic structure of the PdSe_2_ thin films. A low‐magnification TEM image of the PdSe_2_ thin film transferred onto an ultrathin carbon support film is shown in Figure 1b. Figure 1c shows a typical experimental atomic‐resolution HAADF‐STEM image of PdSe_2_ along the [001] crystallographic axis. The atomic‐resolution image in the top‐left corner indicates an orthogonal lattice devoid of noticeable crystal defects. The lattice plane spacing is 0.29 nm, corresponding to the PdSe_2_ (200) lattice plane, which is consistent with previous literature reports.^[^ ^29^ ^]^ Figure 1d shows the selected area electron diffraction (SAED) pattern of PdSe_2_. The clarity and orderly arrangement of the diffraction spots suggest a single‐crystalline structure in PdSe_2_. Additionally, energy dispersive spectroscopy (EDS) was conducted to characterize the elemental composition and distribution of the PdSe_2_ thin film. Figures 1f,g present the EDS elemental mapping, revealing a uniform distribution of Pd and Se across the PdSe_2_ thin film transferred onto SiO_2_/Si. Moreover, the EDS spectrum exhibits sharp peaks for Pd and Se, with an atomic ratio of ≈1:1.96 (Figure S1, Supporting Information), confirming that our sample is stoichiometric PdSe_2_.

The phonon vibrations and interlayer coupling properties of the mechanically exfoliated PdSe_2_ flakes were characterized through Raman spectroscopy. As shown in Figure 1e, the characteristic Raman peaks located at ≈146.8, 222.4, and 257.0 cm^−1^ are attributed to the A_g_^1^, B_1g_^2^ and A_g_^3^ vibrational modes in bulk PdSe_2_, respectively. These findings are in line with previous conclusions in the literature.^[^ ^30^ ^]^ As the material thickness decreases, these three vibrational modes each exhibit a remarkable blueshift, attributable to strong interlayer coupling and hybridization within PdSe_2_.^[^ ^22^ ^]^ Consequently, through gold‐assisted exfoliation, high‐quality, ultrathin, large‐area 2D PdSe_2_ single crystals were successfully prepared.

### 2.2 Interlayer Frictional Characteristics of the 2D Heterostructures

**Figure 2:**
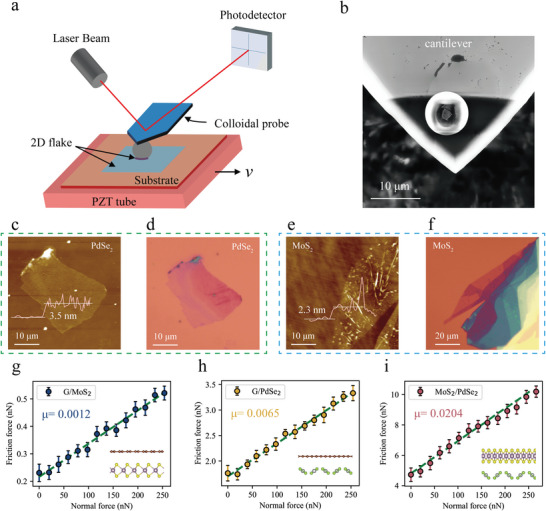
Characterization of interlayer superlubricity in heterostructure systems. a) The experimental arrangement, depicted in a schematic diagram, demonstrates the methodology for measuring the friction between heterostructure layers. The setup involves a substrate securely mounted on an AFM stage, which incorporates a piezoelectric ceramic transducer (PZT) and a Si/SiO_2_ surface. The frictional characteristics of 2D material layers under an applied load are quantified using a custom‐made AFM colloidal probe that exerts a normal force. Furthermore, an objective lens is attached to the AFM head, enabling in situ tracking of the motion of the microsphere probe relative to the substrate. b) SEM top view of the homemade colloidal probe. c) AFM image of PdSe_2_ flakes on a Si/SiO_2_ substrate. The inset depicts the height profile. d) Corresponding optical image in c. e) An AFM image showing MoS_2_ flakes positioned on a Si/SiO_2_ substrate is presented. The accompanying inset illustrates the height profile. f) Corresponding optical image in e. g–i) Three different heterostructure combinations, namely, G/MoS_2_ g), G/PdSe_2_ h), and MoS_2_/PdSe_2_ i), were studied with friction as a function of the applied normal load. The figure includes a green dashed line, which serves as the fitting line. The slope of this line signifies the friction coefficient of the respective heterostructure. The illustration represents the associated structural model. Standard deviations define the error bars in this context.

To elucidate the interlayer frictional properties of three heterogeneous junction systems, namely, G/MoS_2_, G/PdSe_2_, and MoS_2_/PdSe_2_, the lateral force module of an AFM was employed for measurement, as depicted in **Figure** **2**a. The experimental apparatus comprises an AFM tip, laser, and PZT tube, with further details concerning the setup furnished in the Methods section. Figure 2b shows a scanning electron microscopy (SEM) image of the colloidal probe assembly. These measurements were performed using the lateral force mode of an AFM instrument, maintaining a constant sliding velocity of 2 µm/s. For friction tests conducted in ambient air, three combinations of sliding materials were utilized: G/MoS_2_, G/PdSe_2_, and MoS_2_/PdSe_2_. In each pair, the first material listed is adhered to the microsphere, and the second material indicates the substrate.

The experimental setup utilized AFM paired with colloidal probes, which were coated with 2D materials. The process of preparing these colloidal probes involves an adhesive bonding technique. This method is akin to that reported by Tang et al.,^[^ ^31^ ^]^ but with the adoption of a high‐strength adhesive and a custom‐made micromanipulator in our approach. This modification enhances the efficiency and precision of probe fabrication. For further details, please refer to the Methods section. Two types of colloidal probes were prepared, namely, graphene colloidal probes and MoS_2_ colloidal probes. SEM images indicate that 2D materials adhere well to the surface of the colloidal probes and exhibit good flatness (Figure S2, Supporting Information).

Optical microscopy was used to assess the dimensions and consistency of the 2D crystals after mechanical exfoliation, as depicted in Figure 2c–f and Figure S3 (Supporting Information). In all instances, a linear increase in the average friction force was observed with increasing normal load, as illustrated by the fitted lines in Figure 2g–i, where the slopes are clearly labelled. This linear correlation between the friction force and normal load mirrors the conventional macroscopic friction law. A comparative analysis of different friction pairs revealed variations not only in the absolute values of frictional forces but also in the friction coefficients (μ) derived from the fitted lines. For instance, the friction coefficient for a MoS_2_‐coated microsphere probe sliding on a PdSe_2_ substrate (MoS_2_/PdSe_2_) was found to be 0.0204, as shown in Figure 2i. However, when the microsphere probe is coated with graphene and slides on MoS_2_ and PdSe_2_ substrates, the friction coefficients are significantly lower than those of the MoS_2_/PdSe_2_ system, which are 0.0012 and 0.0065, respectively, both of which are in the superlubricity regime. The typical friction loop curves for the three systems can be found in Figure S4 (Supporting Information). The region enclosed by the forward and reverse curves represents the energy dissipated due to friction at the nanoscale.

Notably, frictional forces are detectable even at zero or negative applied loads, suggesting the impact of adhesive forces on frictional behavior. Within the context of JKR theory,^[^ ^32^ ^]^ adhesion that varies with load is a critical aspect of the model. In instances where a linear correlation emerges between the force of friction and the applied load, adhesion is commonly interpreted as a counteracting force. The literature reports^[^ ^33^ ^]^ that the friction‐load relationship within the system can be approximately described by a linear formula *F_L_* =  μ(*F_n_* + *F_ad_*), where *F_n_* denotes the normal load and *F_ad_* denotes the adhesion force. The frictional force observed when there is no applied load is associated with the adhesion force between the material adhered to the colloidal probe and the substrate. It is worth noting that estimating the adhesive force of the system through linear formulas lacks accuracy. In this study, when the normal load is zero, the shear forces in all three systems are positive. This result clearly indicates that the frictional resistance observed under zero load conditions is primarily attributed to interlayer adhesion.

### 2.3 Adhesion Measurement

**Figure 3:**
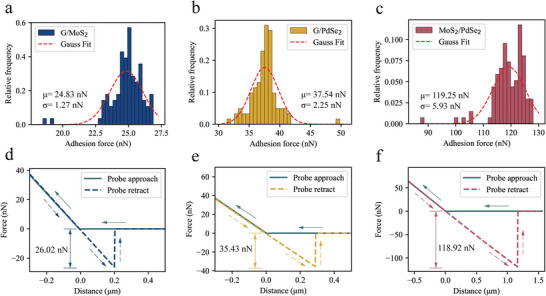
Measurements of the interlayer cohesive force. a–c) The adhesive force histograms between the tip and the 2D materials were measured for the G/MoS_2_ a), G/PdSe_2_ b), and MoS_2_/PdSe_2_ c) heterostructure systems. The red dashed line represents the Gaussian fitting curve. d–f) Typical force‒distance curves (sometimes known as F‒D curves) recorded on the complete heterostructure interfaces of G/MoS_2_ d), G/PdSe_2_ e), and MoS_2_/PdSe_2_ f) crystals.

Superlubricity is significantly influenced by contact size and interfacial interactions, as emphasized in the literature.^[^ ^34^ ^]^ Furthermore, to elucidate the role of interfacial interactions in the experiments, measurements of the interlayer adhesion forces were conducted for the three different heterostructure systems. **Figure** **3**a–c depicts the distribution of the measured adhesion forces for these systems. The data exhibit strong agreement with a Gaussian distribution, yielding mean values of ≈24.83 ± 1.27 nN for $F_{G/MoS_{2}}\;$, 37.54 ± 2.25 nN for $F_{G/PdSe_{2}}\;$, and 119.25 ± 5.93 nN for $F_{MoS_{2}/PdSe_{2}}\;$. The results indicate that the adhesive forces of the heterostructure systems are $F_{G/MoS_{2}}$ < $F_{G/PdSe_{2}}$ < $F_{MoS_{2}/PdSe_{2}}\;$. This is consistent with the trend in the friction coefficients measured for the heterostructure systems. Similarly, Buzio et al. reported that the establishment of a graphene transfer layer marks a shift from a “high friction” to an “ultralow friction” state for the initial colloidal probe. This transition is accompanied by a simultaneous decrease in adhesive forces at the contact interface.^[^ ^35^ ^]^

vdW interactions play a pivotal role in the transfer of stress within and between 2D interfaces, significantly influencing the performance of these interfaces. Generally, dispersion forces are the predominant type of intermolecular force. However, for atoms with high polarity, orientation forces become significant. As evident from Figure 3, the MoS_2_/PdSe_2_ system exhibits an interlayer adhesion that is four times greater than that of the other systems, indicating a greater degree of electron sharing between layers in the MoS_2_/PdSe_2_ system, as confirmed by the charge density difference. The model settings and detailed calculation results are presented in Figures S5 and S6 (Supporting Information), respectively. The charge density, Δρ, illustrates the impact of interface formation on the charge distribution, validating the presence of induced dipoles between the contacting surfaces. The interlayer polarization strength depends on the spatial variation in interlayer charge transfer.^[^ ^36^ ^]^ A detailed analysis of charge transfer in these systems was carried out to more accurately reveal the phenomenon of interlayer polarization. Through quantitative calculations and visual analysis, it was found that in the G/MoS_2_ system (Figure S7a, Supporting Information), there is almost no noticeable charge transfer, with electron gains and losses primarily confined within the MoS_2_ layer. Notably, in the G/PdSe_2_ system, the amount of charge transfer is minimal, which also accounts for the relatively low interlayer friction and adhesion observed in this system. In the MoS_2_/PdSe_2_ system, more significant interlayer charge transfer was observed, as evidenced by the formation of positive and negative charge centers, indicating strong interlayer polarization in this system. Refer to Figure S7 (Supporting Information) for detailed information.

### 2.4 Layer Thickness Effect on the Interfacial Frictional Performance

**Figure 4:**
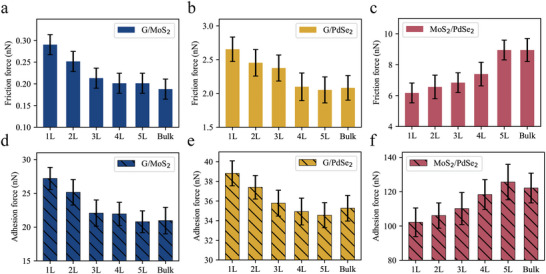
Variational trends of interfacial friction and adhesion forces with layer thickness. a–c) The evolution trend of the interlayer frictional force with respect to the substrate thickness in heterostructure systems G/MoS_2_ a), G/PdSe_2_ b), and MoS_2_/PdSe_2_ c). d–f) The trend of the interfacial adhesion force with respect to the substrate thickness in heterostructure systems G/MoS_2_ d), G/PdSe_2_ e), and MoS_2_/PdSe_2_ f).

Using AFM under ambient conditions, the thickness‐dependent friction of three heterostructure systems, G/MoS_2_, G/PdSe_2_, and MoS_2_/PdSe_2,_ was studied, as shown in **Figure** **4**a–c. The thickness of the substrate layers was determined using the tapping mode of AFM. For the G/MoS_2_ and G/PdSe_2_ systems, the friction force initially decreased and then stabilized. In contrast, the interlayer friction force of MoS_2_/PdSe_2_ shows the opposite trend, where the friction force increases with increasing layer thickness. With the increasing number of substrate material layers, both the friction force and adhesion force are influenced, showing a consistent trend of change. This suggests that the thickness‐dependent friction at the interface of the heterostructure is connected to the vdW interaction forces between the interlayers.

### 2.5 Rotational Anisotropic Friction

**Figure 5:**
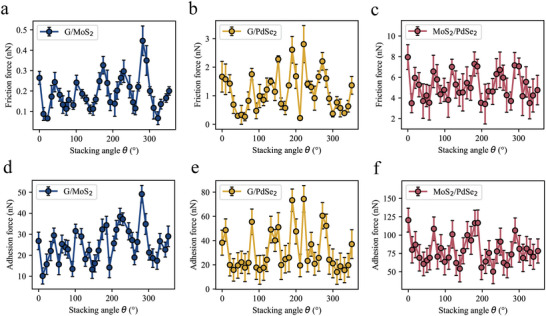
The measured friction of heterostructures exhibits rotational anisotropy. a–c) The evolution of interlayer friction in heterostructure systems G/MoS_2_ a), G/PdSe_2_ b), and MoS_2_/PdSe_2_ c) with stacking angle, as measured by AFM. d–f) The evolution of the interlayer adhesion force in heterostructure systems G/MoS_2_ d), G/PdSe_2_ e), and MoS_2_/PdSe_2_ f) as a function of stacking angle.

Frictional anisotropy is a distinctive and intrinsic feature observed on surfaces with uneven or asymmetric topographies. In 2011, Choi et al. conducted the initial experimental investigation into the unique frictional properties of monolayer graphene obtained from a silica surface using friction force microscopy (FFM).^[^ ^37^ ^]^ Their angle‐dependent friction measurements revealed a 180° periodicity in friction within each domain, which they ascribed to the anisotropic ripples present in graphene. In essence, the varying angles between the scanning direction and the ripples resulted in varying degrees of out‐of‐plane deformation in the monolayer graphene. This phenomenon of frictional anisotropy due to probe scanning along different crystallographic directions is distinct from the rotational anisotropy caused by interlayer stacking angles. To explore how relative rotation angles affect the superlubricity of 2D heterostructures, the substrate was systematically rotated, altering the relative orientation between the material‐coated tip and the substrate. During this process, the average frictional force between the heterostructures at various rotation angles was recorded. The rotational anisotropy of three distinct heterostructures was quantified at intervals of 10 degrees, as illustrated in **Figure** **5**a–c and Figure S8 (Supporting Information). The average frictional force correlates with the rotation angle of the wrapped tip as it slides on the substrate material, displaying a frictional characteristic symmetry of ≈60 degrees rotationally. This rotational symmetry is a common characteristic of materials such as graphene and MoS_2_, effectively emphasizing the hexagonal structure of the material lattice adhering to the colloidal probe. It provides a representation of the contact states between the layers at these particular rotation angles. The presence of low frictional forces suggests that the crystal faces of the heterostructure are not aligned with each other, a finding that aligns with earlier experiments conducted by Dienwiebel et al.^[^ ^8^ ^]^ The anisotropy observed in heterostructures, particularly in the G/MoS_2_ and G/PdSe_2_ systems, remains inconsistent, even when the contact configurations are aligned. This inconsistency is attributed to the mismatch in the lattice vectors between the two surfaces. Consequently, in such scenarios, the orientation‐dependent frictional anisotropy can be considered negligible, irrespective of the differences in interfacial orientation.^[^ ^12^ ^]^ Moreover, while recording the average frictional force between heterostructures at each rotation angle, the interlayer adhesion forces at the current rotation angle were also documented, as shown in Figure 5d–f. Adhesion and friction exhibit similar variation patterns with changes in the stacking angle; namely, high interlayer friction corresponds to a high adhesion force. This similarity suggests that there may be a common underlying mechanism affecting the variation between these two forces, which is the change in interlayer interactions. The lattice mismatch at different stacking angles at heterostructure interfaces and the resulting moiré patterns lead to an uneven distribution of interlayer charges or electron cloud density. This change in distribution forms dipoles, leading to interlayer polarization, which in turn directly impacts the interlayer adhesion and friction forces.

### 2.6 Velocity Effect on Interlayer Friction

**Figure 6:**
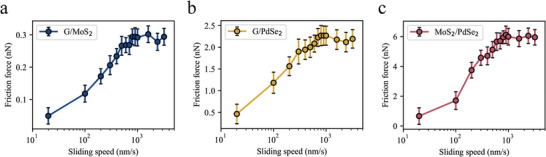
The velocity‐dependent nature of the interfacial friction force. a–c) The variation in interlayer friction in the heterostructure systems G/MoS_2_ a), G/PdSe_2_ b), and MoS_2_/PdSe_2_ c) as a function of scanning speed, measured using AFM.

To explore the velocity dependence of friction, the effect of changing the scanning frequency while maintaining a constant scanning size of 500 nm was examined. The interlayer frictional forces for the three heterostructure systems, G/MoS_2_, G/PdSe_2_, and MoS_2_/PdSe_2_, were plotted as a function of sliding speed on a logarithmic scale, as depicted in **Figure** **6**. The study indicates that under all the investigated normal forces (50 nN), the frictional forces of these monolayer materials exhibit logarithmic growth with increasing sliding speed. The results also show that frictional forces generally stabilize when the speed exceeds 1 µm/s. These patterns align with the thermally activated Prandtl–Tomlinson (PT) model, which posits that frictional forces increase with sliding speed due to the influence of thermal energy.^[^ ^38^ ^]^ According to the PT model, two distinct regimes can be readily discerned in terms of how frictional force changes with sliding speed. The first regime exhibits a logarithmic increase in frictional force at sliding speeds below a critical speed, while the second regime maintains a stable level of frictional force at sliding speeds surpassing this critical value. Therefore, in accordance with the PT model, the observed critical speed of ≈1 µm/s can be regarded as the speed at which all three heterostructure systems attain a steady state of frictional forces. Similar velocity‐dependent frictional characteristics have been previously observed in various materials, such as mica^[^ ^38^ ^]^ and graphite.^[^ ^39^ ^]^

### 2.7 Machine Learning Potential Fitting

Recently, machine learning potentials (MLPs) have offered a new paradigm for research.^[^ ^40^ ^]^ MLPs enhance MD simulations by improving efficiency, scalability, and accuracy, thus allowing for more comprehensive studies of complex chemical and material systems. For the three heterostructure systems discussed in this paper, the absence of interatomic potentials poses a substantial challenge in providing a clear atomic‐level physical understanding. To address this issue, there is an intention to develop a neuroevolutionary potential (NEP) model,^[^ ^41^ ^]^ based on datasets computed through density functional theory (DFT). This model will facilitate MD studies focused on interlayer friction.

The dataset under discussion encompasses a total of 2828 structures. These structures are systematically extracted to create the testing dataset, resulting in the final composition of the training and testing datasets containing 369 and 2459 structures, respectively. The dataset is divided into three main parts. First, the first part includes additional geometric structures obtained from ab initio molecular dynamics (AIMD) simulations. Specifically, we simulated at a temperature of 300 K and employed a Nosé‒Hoover thermostat to ensure temperature stability. The total simulation time was 50 picoseconds, with an integration time step of 1 *fs*, and system configurations were sampled every 0.5 ps. Second, the second part covers the initially designed bulk and heterostructure systems. In these structures, we introduced a strain range of −5% to +5% and random atomic position offsets of up to 0.1 Å to simulate stress and structural distortions that may occur under nonideal conditions. Finally, the third part involves running molecular dynamics simulations using a preliminary trained NEP model to generate various structural configurations of the systems. A subset of these configurations was selected for DFT calculations, which were then used for further training and optimization of the NEP model. In each training generation or iteration, the NEP model calculates the energy, the forces acting on each atom, and the nine‐component virial values for every structure within the dataset. These computed results are then juxtaposed with the corresponding values obtained from DFT calculations to verify and ensure computational accuracy.

**Figure 7:**
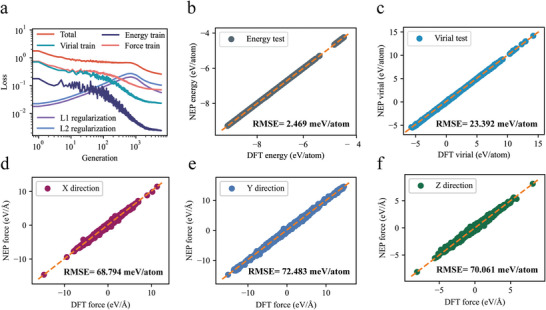
Training process for the NEP machine‐learned potential. a) The evolution of the loss function concerning the training dataset throughout the generative process. b,c) A comparison is made between the predictions derived from the NEP and the reference data from DFT for energy and virial properties in the test datasets. d–f) An evaluation is performed to assess the agreement between the NEP predictions and DFT reference data for the testing dataset along the x‐, y‐, and z‐axes.

**Figure** **7**a illustrates the progression of various loss functions throughout the NEP training process, encompassing a total of 600 000 iterations. The definition of the loss function is detailed in Note 2 (Supporting Information). As the training progresses through generations, a general trend of decreasing and converging root mean square errors (RMSEs) is observed for the energy, force, and virial loss functions, despite some initial fluctuations. Specifically, in Figure 7b,c, the converged RMSEs for energy and virial on the test dataset are recorded at 2.469 and 23.392 meV atom^−1^, respectively. Regarding the forces in the x, y, and z directions, the converged RMSE values are detailed in Figure 7d–f as 68.794, 72.483, and 70.061 meV atom^−1^, respectively. These values suggest that the trained NEP model achieves a high degree of accuracy, comparable to the results obtained from DFT calculations.

### 2.8 Verification of the NEP Prediction Accuracy

**Figure 8:**
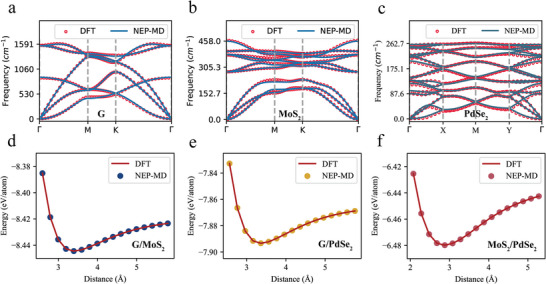
Precise validation of the NEP machine learning potential. a–c) An assessment of the phonon spectra of graphene a), MoS_2_ b), and PdSe_2_ c) materials involving a comparison between the predictions obtained from the NEP and the reference data derived from DFT. d–f) An examination of the energy evolution with interlayer spacing in the three heterostructure systems, namely, G/MoS_2_ d), G/PdSe_2_ e), and MoS_2_/PdSe_2_ f).

The NEP‐based simulation is carried out in a 10 × 10 × 1 supercell. As shown in **Figure** **8**a–c, to verify the effectiveness of the NEP, the phonon dispersion curves of the materials were obtained through the finite element method. Compared with DFT, the NEP can accurately replicate phonon dispersions with exceptional precision, especially in the low‐frequency region. Both approaches indicate the absence of soft modes (imaginary frequencies) in the phonon dispersion curves, suggesting the thermodynamic stability of all the materials. Additionally, for the three heterostructure systems (G/MoS_2_, G/PdSe_2_, and MoS_2_/PdSe_2_), energy calculations at different interlayer spacings were conducted to compare the disparities between the DFT and NEP predictions, as presented in Figure 8d–f. The results demonstrate that the NEP effectively captures the impact of the interlayer spacing on the system energy. In conclusion, for the study of these three heterostructure systems, our machine learning potential proves to be both effective and accurate.

### 2.9 MD Simulation of Interlayer Friction

To clarify the fundamental causes of directional friction occurring at the heterointerface and to comprehend the variations in the underlying mechanisms, MD simulations were utilized to investigate the interlayer friction characteristics of the heterostructures. The model system in our study consists of a flexible slider and a rigid substrate. To replicate the compliance of an AFM cantilever, the flexible slider was connected to a virtual atom using a spring mechanism to facilitate its motion. During the simulated sliding, a uniform normal load (*F_n_* =  0.1 nN/atom) is applied to the slider, and the virtual atom propels the slider in the x‐direction at a constant velocity (*v*  =  10 m/s) with a spring constant of *k*  =  30.0 N/m.^[^ ^42^ ^]^ Three distinct MD models of heterostructure systems were crafted: G/MoS_2_ (88.7 Å × 76.8 Å), G/PdSe_2_ (68.7 Å × 93.9 Å), and MoS_2_/PdSe_2_ (113.9 Å × 82.2 Å). Periodic boundary conditions were enforced in the x and y directions for the substrate, while free boundary conditions were maintained in the z direction. The simulation was conducted within the NVT ensemble, employing a Nosé‒Hoover thermostat to regulate the system's temperature at 300 K. A timestep of 1 *fs* was utilized for the simulations, and all computations were executed using the LAMMPS package.^[^ ^43^ ^]^

**Figure 9:**
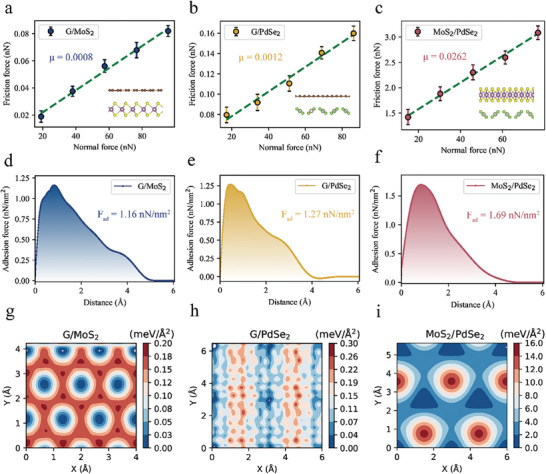
MD simulations of interlayer friction performance. a–c) Frictional force as a function of normal force for G/MoS_2_ a), G/PdSe_2_ b), and MoS_2_/PdSe_2_ c). The corresponding friction coefficients, μ, are indicated. d–f) Adhesion force profiles against separation distance, with peak forces, F_ad_, highlighted. g–i) PES for each heterostructure system, with color gradients signifying energy variations from low (blue) to high (red).

The displayed friction force represents the time‐averaged force exerted on virtual atoms along the sliding path. Simulations of the same sliding direction were conducted multiple times (five times). **Figure** **9**a–c depicts the relationship between the interfacial friction and normal load in the three heterostructure systems. This relationship exhibits a distinct linear pattern, aligning with the common characteristics of solid friction. Notably, the G/MoS_2_ and G/PdSe_2_ heterostructures exhibit ultralow friction coefficients of 0.0008 and 0.0012, respectively, indicating their excellent superlubricity. In contrast, the friction coefficient for the MoS_2_/PdSe_2_ combination is relatively higher at 0.0262, suggesting the possible presence of more complex interlayer interactions leading to greater friction. Additionally, in the MoS_2_/PdSe_2_ system, the change in interlayer spacing during the sliding process was significantly greater than that in the other systems, as detailed in Figure S9 (Supporting Information). By steadily lifting the slider at a rate of *v*  =  0.01 Å/ps and monitoring the overall interface force and interlayer relative displacement, the results are presented in Figure 9d–f. The adhesive force relationship for the three systems is $F_{G/MoS_{2}}$ < $F_{G/PdSe_{2}}$ < $F_{MoS_{2}/PdSe_{2}}\;$, which aligns with the conclusions drawn from the experimental section in the preceding chapters. By examining potential energy surfaces (PESs), more intuitive insight into the structural characteristics of the surfaces of these materials was gained. The PES was obtained using a grid‐like method similar to that used for static calculations. For the G/MoS_2_ and G/PdSe_2_ systems, the potential energy fluctuations are merely 0.2 and 0.3 meV/Å^2^, respectively, which are significantly lower than those in the MoS_2_/PdSe_2_ heterostructure. This phenomenon corresponds to their higher friction coefficients and adhesion forces. Moreover, these findings are consistent with the research reported by Vazirisereshk^[^ ^44^ ^]^ and Wang^[^ ^45^ ^]^ et al., who noted that the G/MoS_2_ system possesses an extremely smooth potential energy surface, thereby exhibiting lower sliding resistance.

**Figure 10:**
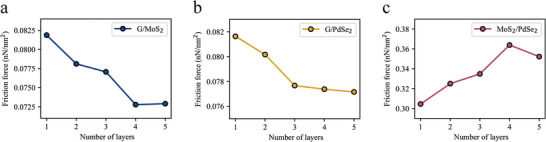
Variation in friction with substrate layer thickness. a–c) Variation in frictional force with layer number in heterostructure systems G/MoS_2_ a), G/PdSe_2_ b), and MoS_2_/PdSe_2_ c).

Additionally, the relationship between the interlayer friction and variations in the substrate thickness for the three heterostructure systems was investigated, with the bottommost layer of the substrate being firmly anchored. As illustrated in **Figure** **10**, for G/MoS_2_ and G/PdSe_2_, the interlayer friction force decreases as the thickness of the substrate increases. In contrast, the MoS_2_/PdSe_2_ heterostructure system exhibits the opposite trend, which aligns well with the experimental results. Furthermore, beyond a specific threshold of layers, approximately four layers, the number of layers ceased to significantly impact the friction force. This observation implies that when the substrate consists of fewer layers, the interactions between these layers likely play a pivotal role in modulating the friction force. However, as the number of substrate layers surpasses a certain threshold, the influence of layer count on friction tends to diminish, suggesting that other factors may become more predominant in determining friction behavior.

**Figure 11:**
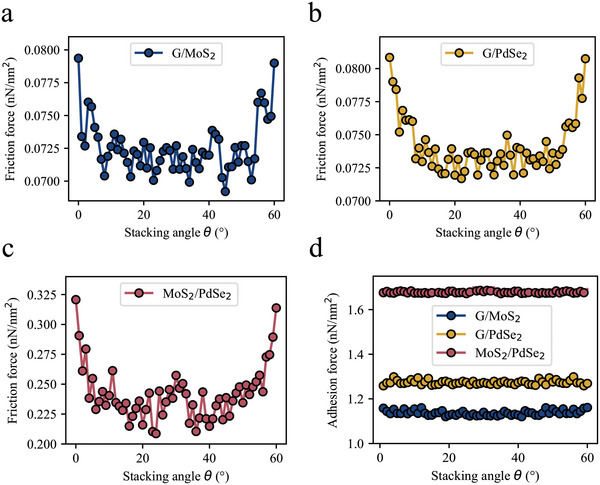
Interlayer friction and adhesion force as a function of stacking angle. a–c) Variation in frictional force as a function of stacking angle in heterostructure systems G/MoS_2_ a), G/PdSe_2_ b), and MoS_2_/PdSe_2_ c). d) Trend of adhesive force with changes in stacking angle.

Using MD techniques, the evolution of the interlayer friction force under various stacking angles for the three heterostructure systems was investigated. The findings are presented in **Figure** **11**a–c. All three heterostructure systems exhibit a 60‐degree periodicity (sixfold symmetry), which aligns well with the hexagonal nature of the slider material lattice. For the G/MoS_2_ and G/PdSe_2_ heterostructure systems, the variation in frictional force with twist angle is minimal. In this scenario, smooth sliding can occur irrespective of the orientation angle of the interface, resulting in friction anisotropy that is practically negligible. In contrast, the MoS_2_/PdSe_2_ heterostructure system experiences a broader fluctuation in friction force with the change in stacking angle, suggesting that much lower frictional resistance can be attained at large incommensurate contact states than at aligned configurations, thereby exhibiting pronounced angle‐dependent anisotropic friction. Furthermore, the adhesive forces at different twist angles were calculated. As depicted in Figure 11d, the adhesive forces for the three heterostructure systems remain stable at various stacking angles. This indicates that the variation in frictional force at different stacking angles is mainly caused by the commensurate nature of the interface lattice, which affects the sliding resistance within the interface plane rather than being dominated by adhesion forces. This finding is consistent with conclusions reported in previous literature, where no significant correlation between the interlayer shear stress and twist angle was observed.^[^ ^46^ ^]^

## 3 Discussion

The total interlayer interactions *E_total_* of 2D materials consist of dispersion interactions *E_LDF_* and nondispersion interactions (polar interactions). Gudarzi and colleagues discovered an approximately linear relationship between the interlayer dispersion interactions and the total interlayer interactions:^[^ ^47^ ^]^

(1) $$\frac{E_{LDF}}{E_{total}}\approx 1-f$$

where *E_LDF_* represents the contribution of dispersion terms to vdW interactions. Here, *f* is Pauling's ionicity, which provides a fairly accurate polarity scale for layered crystal materials. *f* can be calculated using the following equation:^[^ ^48^ ^]^

(2) $$f=1-exp^{-{\left(\frac{X_{A}-X_{B}}{2}\right)}^{2}}$$

where *X_A_* and *X_B_* are the electronegativities of elements A and B, respectively. Clearly, according to Equation (1), the smaller the degree of ionicity is, the greater the influence of the dispersion force.

The dispersion interactions *E_LDF_* can be calculated based on Hamaker theory.^[^ ^49^ ^]^ The potential energy per unit area, *E_LDF_*, between two plates of thickness *h* and separated by distance *D* is calculated as follows:^[^ ^50^ ^]^

(3) $$\begin{array}{c} E_{LDF}=-\frac{H}{12\pi }\left[\frac{1}{{\left(D-d_{0}\right)}^{2}}+\frac{1}{{\left(D-d_{0}+2h\right)}^{2}}-\frac{2}{{\left(D-d_{0}+h\right)}^{2}}\right] \end{array}$$

The *d*_0_ term is defined so that the proposed equation follows the vdW energy distribution.^[^ ^47^ ^]^ *H* is the Hamaker constant, typically ranging from (0.4 −4) × 10^−19^ J. For simple models involving material self‐interaction,^[^ ^51^, ^52^ ^]^ the estimation can be made using a few parameters according to the Lifshitz theory:^[^ ^53^ ^]^

(4) $$H=\left\{\begin{array}{cc} \frac{3}{16\sqrt{2}}\hbar \omega_{b}\frac{{\left(n^{2}-1\right)}^{2}}{{\left(n^{2}+1\right)}^{3/2}}, & semiconductor\\ \frac{3}{16\sqrt{2}}\hbar \omega_{p}, & Drude\;metal\\ \frac{3}{4\pi }\hbar \omega_{d}, & Graphene \end{array}\right.$$

The parameters in the above formulas include the reduced Planck constant ℏ, the refractive index *n*, the bandgap ℏω_*b*_ of the semiconductor, the plasma frequency ω_*p*_ of the metal, and the height ℏω_*d*_ of the Dirac cone in graphene.

The Hamaker constants for different materials can be estimated using equation (4). According to a recent publication,^[^ ^51^ ^]^ the Hamaker constants for graphene and MoS_2_ are 1.3 and 0.8 eV, respectively. The refractive index *n* for PdSe_2_ is reported to be 3.9045,^[^ ^54^ ^]^ and its measured optical bandgap ℏω_*b*_ is 1.43 eV.^[^ ^55^ ^]^ Substituting these values into formula (4), the Hamaker constant for PdSe_2_ is calculated to be 0.588 eV. When determining the Hamaker constant for a heterostructure system, the Hamaker constant between two different materials is given by the geometric mean of their respective self‐interaction constants.^[^ ^52^, ^56^ ^]^ *H* ^(*aa*)^ represents the Hamaker constant for one material, *H* ^(*bb*)^ corresponds to another material, and *H* ^(*ab*)^ represents the cross‐interaction Hamaker constant between the two materials.

(5) $$H^{\left(ab\right)}\approx \sqrt{H^{\left(aa\right)}H^{\left(bb\right)}}$$

In this study, we assume that the thickness of the two plates is ≈2.5 Å, with an interlayer distance of 3.4 Å between them. Based on this assumption, we can accurately calculate the interlayer dispersion forces within the system. For the G/MoS_2_ system, the corresponding mixed Hamaker constant *H*_1_ is 1.020 eV. In the G/PdSe_2_ system, the corresponding mixed Hamaker constant *H*_2_ is 0.874 eV. For the MoS_2_/PdSe_2_ system, the corresponding mixed Hamaker constant *H*_3_ is 0.686 eV. Substituting these parameters into equation (3), the approximate contributions of the dispersion forces per unit area for the G/MoS_2_, G/PdSe_2_, and MoS_2_/PdSe_2_ systems are calculated to be 16.781, 14.379, and 11.286 meV, respectively. Using first‐principles calculation methods, we obtained the total binding energy per unit area for the three systems. The binding energies per unit area for the three systems are 19.130, 16.867, and 25.543 meV, respectively. This variance underscores the contribution of nondispersion interactions (primarily polar interactions) in 2D materials.

Based on the above data, the following analysis can be performed:

For the G/MoS_2_ system, dispersion interactions contribute 87% of the total energy, and polar interactions contribute 13%. In the G/PdSe_2_ system, dispersion interactions accounted for 85% of the total interactions, and polar interactions accounted for 15%. In the MoS_2_/PdSe_2_ system, dispersion interactions constitute 44% of the total interactions, while polar interactions contribute 56%. According to previous experimental results, the interlayer friction increases with increasing adhesion force, showing a positive correlation. However, the absolute values of dispersion interactions are not significantly different across the three systems (16.781, 14.379, and 11.286 meV, respectively), indicating that differences in adhesion force among these systems primarily arise from variations in nondispersion (polar) interactions. Combined with the experimental results on the friction force, it is shown that if polar interactions predominate in the interlayer interactions, the interlayer friction force is greater. The calculation results reveal that the G/MoS_2_ and G/PdSe_2_ systems have a smaller proportion of polar interactions, whereas the MoS_2_/PdSe_2_ system has the largest proportion of polar interactions, which is consistent with the experimental findings showing lower interlayer friction in the G/MoS_2_ and G/PdSe_2_ systems and greater friction in the MoS_2_/PdSe_2_ system.

According to equation (1), the ionicity *f* of a material is closely related to its polar interactions; the smaller f is the smaller the proportion of polar interactions in total interactions. Based on the literature cited in the literature^[^ ^47^ ^]^ and the results of our calculations, the ionicity *f* of graphene is 0, that of MoS_2_ is 0.043, and that of PdSe_2_ is 0.030. The friction force is smaller in the G/MoS_2_ and G/PdSe_2_ systems and greater in the MoS_2_/PdSe_2_ system, suggesting that the magnitude of the interlayer friction force is associated with the differences in ionicity between the two materials at the friction interface. The ionicity difference is 0.043 between graphene and MoS_2_, 0.030 between graphene and PdSe_2_, and 0.013 between MoS_2_ and PdSe_2_. Clearly, the greater the difference in ionicity is, the lower the interlayer friction force. This indicates that the larger the difference in ionicity between the two 2D materials comprising the sliding interface of a heterostructure system is the lower the interlayer friction force. This provides a core criterion for designing ultralow‐friction 2D material friction pairs.

## 4 Conclusions

In conclusion, our findings illuminate the mechanisms by which G/MoS_2_ and G/PdSe_2_ heterostructure systems attain remarkably low friction coefficients, ≈10^−3^. In contrast, the MoS_2_/PdSe_2_ heterostructure interfaces present higher friction coefficients, ≈0.02, primarily as a result of pronounced interfacial interactions induced by significant interlayer charge transfer. This distinction underscores the critical role of the ionic nature of 2D materials in modulating frictional forces, where greater ionicity between materials correlates with reduced friction. Both experimental observations and MD simulations substantiated that in the G/MoS_2_ and G/PdSe_2_ heterostructures, the interlayer friction decreased with increasing layer thickness. Conversely, in MoS_2_/PdSe_2_ systems, friction increases with layer thickness. The influence of the layer thickness on the friction performance stabilizes at approximately four layers and is predominantly influenced by the vdW forces among the heterostructure layers. These findings not only corroborate the pivotal role of low adhesion at the contact interface in achieving ultralow friction performance but also introduce novel insight: the frictional behavior in heterostructure systems is intricately linked to the ionic contrasts between layered materials. This insight provides a strategic criterion for designing advanced materials with optimized tribological properties, enhancing our understanding and application of layered solid lubricants.

## 5 Experimental Section

### Sample Preparation

Graphene and MoS_2_ were obtained through mechanical exfoliation using 3M Scotch tape. Graphene sheets were prepared from highly oriented pyrolytic graphite (HOPG, Institute of Metal Research) and subsequently transferred onto a silicon substrate with an ≈300 nm thick layer of silicon dioxide (Si/SiO_2_). Similarly, monolayer and few‐layer MoS_2_ sheets were mechanically exfoliated from single‐crystal high‐orientation 2H phase MoS_2_ (Taizhou SUNANO New Energy Co., Ltd.). The specific operational steps for the preparation of few‐layer 2D PdSe_2_ samples using the gold‐assisted mechanical exfoliation method are outlined in Note 1 (Supporting Information).

### Sample Characterizations

After visual inspection using an optical microscope (BX53, Olympus), AFM (MFP‐3D, Asylum Research Inc.) was used to determine the thickness of the flakes. A commercial silicon probe (AC240TS‐R3, Olympus, with a nominal spring constant of ≈2 N m^−1^) was utilized for imaging the surface topography. The morphological and elemental mapping data of the prepared samples were analyzed using a focused ion/electron dual‐beam system equipped with an EDS system (FEI Helios G4 CX). Raman spectra of the 2D nanosheets, which were transferred and deposited onto the SiO_2_/Si substrate, were acquired using a confocal Raman microscope (Alpha300R, WITec) under ambient conditions. A 532 nm wavelength laser and a 50x long‐working‐distance objective were employed for the measurements. Prior to data collection, calibration was carried out using a standard silicon wafer. To mitigate the risk of sample damage or laser‐induced heating, all measurements were performed at an incident power level set to 10% of the maximum power output following the completion of all friction tests.

### Preparation of Microsphere Probes

To minimize the significant impact of edges on interlayer friction, a method of wrapping 2D materials around silicon spheres was employed. To fabricate microsphere probes, monodispersed silica microspheres with a diameter of 10 µm were acquired from Suzhou NanoMicro Technology Co., Ltd., along with rectangular cantilevers devoid of tips (TL‐CONT, with a nominal spring constant of ≈0.2 N/m, sourced from NANOSENSORS). Prior to the initiation of the process, a custom micromanipulator, boasting a tip size of ≈10 µm, was installed onto the 2D material transfer system, specifically the E1‐T model produced by MetaTest. This micromanipulator facilitated the precise application of a minimal quantity of epoxy resin adhesive onto the tip‐less end of the cantilever. Next, another clean micromanipulator was used to pick up a pristine silica sphere from a glass slide, align it with the adhesive‐coated cantilever, and meticulously affix the sphere to the cantilever. After the adhesive had been allowed to cure for a period exceeding 24 h, a small additional amount of adhesive was carefully applied to the top of the previously attached silica sphere.

Subsequently, the entire 2D material sheet, which had been prepared on polydimethylsiloxane (PDMS), was introduced into the transfer system. It was carefully pressed against the sphere probe while maintaining precise alignment. Upon initial contact, it was gently pressed to a certain depth to ensure complete contact between the 2D material sheet and the silica sphere. This process served to expel any excess adhesive, resulting in a very thin residual adhesive layer at the interface, which could be disregarded for its impact on the curvature of the 2D material sheet. Finally, the glass slide holding the PDMS was promptly raised to separate the 2D sheet, leaving it securely adhered to the silica sphere. Due to the very high tensile strength (stickiness) of the adhesive, the 2D sheet could be separated from the PDMS even before it was fully dried.

### Measurements of Friction

Friction measurements were conducted using the lateral force mode of MFP‐3D AFM under ambient conditions (room temperature: 23 ± 2 °C, relative humidity: 45 ± 3%). The probe's normal spring constant was calibrated utilizing a noncontact technique,^[^ ^57^ ^]^ and the lateral detection sensitivity of the probe was determined with the assistance of an antiferromagnetic lateral force calibrator.^[^ ^58^ ^]^ The AFM tip exerted pressure on the substrate's upper surface, enabling interlayer sliding via the lateral movement feature of the piezoelectric displacement platform. Under these ambient conditions, friction tests were executed by inducing the sample to slide against the probe as the applied load decreased. The scanning area was set at 1000 nm × 1000 nm, and the scanning rate was set at 1 Hz. Friction forces were computed based on five repeated measurements at each normal load, with the values determined by taking the half difference of lateral force loops acquired during scanning along the same path and returning.

### DFT Calculation Settings

The first‐principles calculations were carried out using the Vienna Ab initio Simulation Package (VASP),^[^ ^59^ ^]^ utilizing DFT. The ion‐electron interactions were modelled using a plane wave basis set and the projector augmented‐wave (PAW) method.^[^ ^60^ ^]^ The exchange‐correlation function was described within the generalized gradient approximation (GGA), employing the Perdew–Burke–Ernzerhof (PBE) functional.^[^ ^60^, ^61^ ^]^ The structural parameters of the initial model are shown in Figure S5, which includes a vacuum space of 20 Å along the Z‐axis. All calculations employed the DFT‐D3 correction method^[^ ^62^ ^]^ to account for interlayer dispersion corrections. The relaxation of the atomic structure continued until all the force components were less than 0.001 eV/Å, and energy convergence was achieved when the energy difference was less than 1.0 × 10^−7^ eV. In the AIMD simulations, the Γ point of the Brillouin zone was exclusively sampled with a k‐point grid density of 1 × 1 × 1. The energy cut‐off was established at 600 eV, and the time step was fixed at 3 fs. The electronic self‐consistency convergence criterion was defined at 10^−5^ eV. For DFT static calculations, the KSPACING parameter was set to 0.15 Å^−1^, with the energy cut‐off remaining at 600 eV.

### Initial Training Dataset Construction

The initial dataset for developing the NEP model was derived from these optimized supercell structures. To construct deformed structures, we applied up to 3% random variations in the lattice vectors and up to 0.1 Å random shifts in atomic positions. Additionally, we created a set of transverse shift configurations by employing either rigid or flexible shifts at the top layer, allowing the out‐of‐plane degrees of freedom for each atom to relax. By performing precise DFT calculations on these various supercell configurations, we generated a rich dataset containing energy, force, and virial information, providing a foundation for training the NEP model.

## Conflict of Interest

The authors declare no conflict of interest.

## Supporting information

Supporting Information

## Data Availability

Data sharing is not applicable to this article as no new data were created or analyzed in this study.
