# Supplementary material for: Interlayer Friction and Adhesion Effects in Penta‐PdSe2‐Based van der Waals Heterostructures
Source: Adv Sci (Weinh). 2024 Jul 4;11(34):2400395. doi: 10.1002/advs.202400395 (PMC11425967; doi:10.1002/advs.202400395)
Supplement: Supplementary file 1 — Supporting Information [file ADVS-11-2400395-s001.docx]

**Supplementary Information for**

**Interlayer Friction and Adhesion Effects in Penta-PdSe_2_-based van der Waals Heterostructures**

**Guoliang Ru^1^, Weihong Qi^1,2^*, Shu Sun^1^, Kewei Tang^1^,
Chengfeng Du^1^, Weimin Liu^1,3^***

^1^ State Key Laboratory of Solidification Processing and Center of Advanced Lubrication and Seal Materials, Northwestern Polytechnical University, Xi’an 710072, China

^2^ Shandong Laboratory of Yantai Advanced Materials and Green Manufacturing, Yantai 265503, China

^3^ State Key Laboratory of Solid Lubrication, Lanzhou Institute of Chemical Physics, Chinese Academy of Sciences, Lanzhou 730000, China

*Correspondence should be addressed to [qiwh216@nwpu.edu.cn](mailto:qiwh216@nwpu.edu.cn) (Weihong Qi) and [wmliu@licp.cas.cn](mailto:wmliu@licp.cas.cn) (Weimin Liu).

Supplementary Figures

| 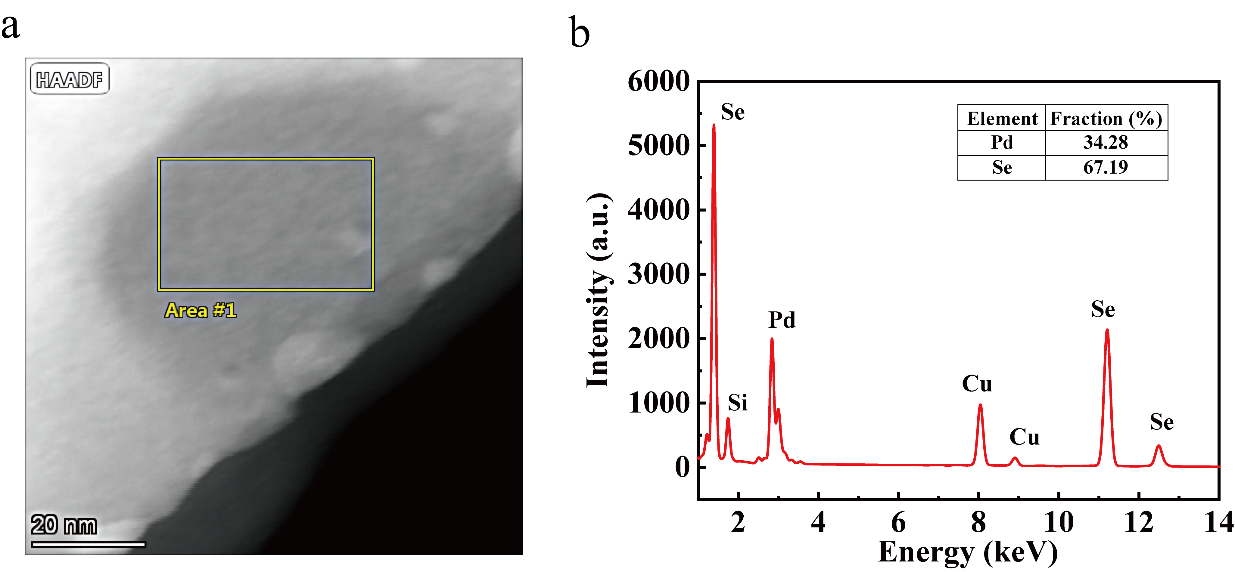 |
| --- |

Figure S1. TEM images of two-dimensional PdSe_2_. An Image of few-layer 2D PdSe_2_ on a grid obtained through low-resolution high-angle annular dark-field scanning transmission electron microscopy (HAADF-TEM). **b** EDS analysis image of few-layer 2D PdSe_2_.

| 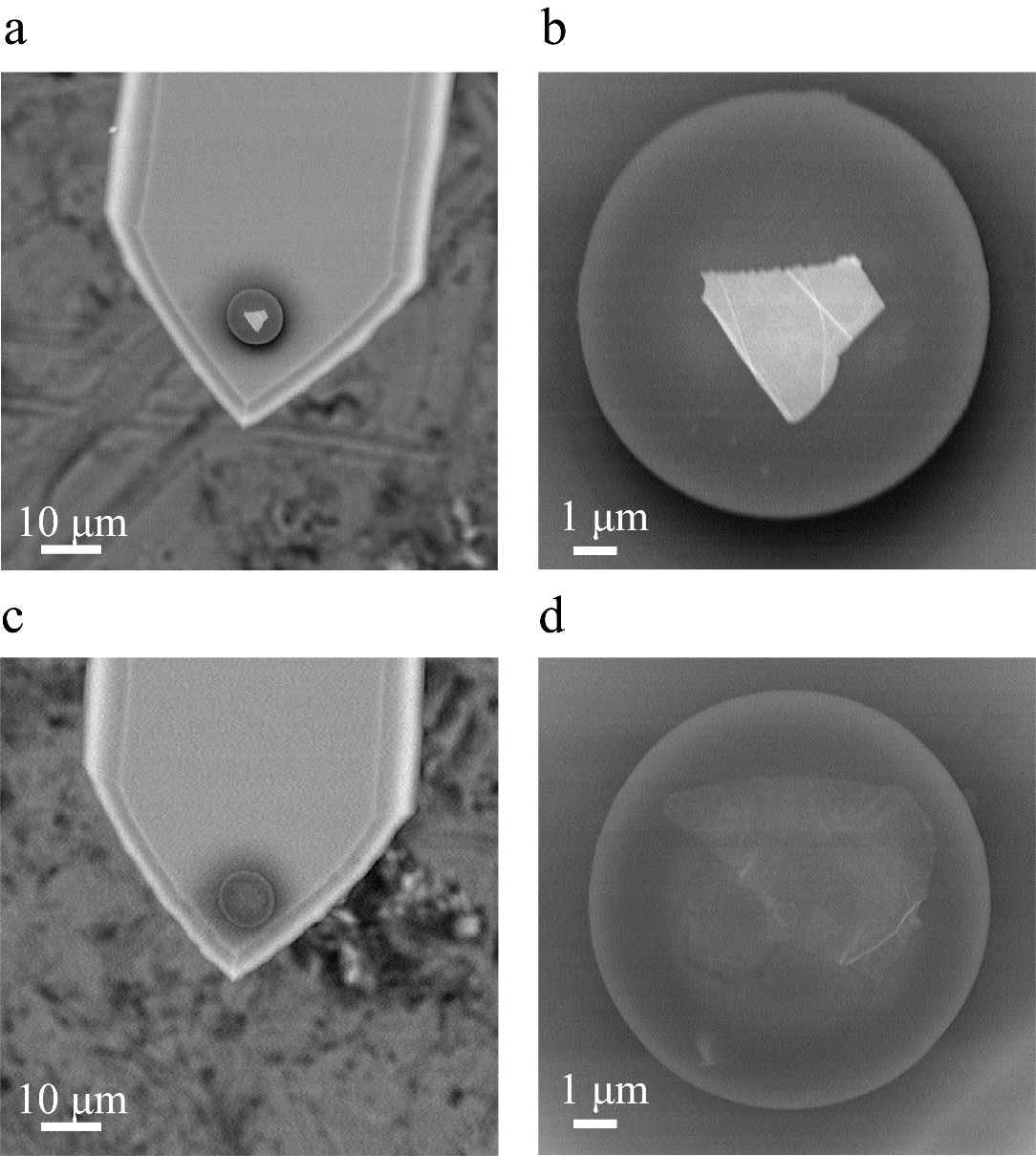 |
| --- |

Figure S2. SEM images of spherical colloidal probes. Top views (a) of colloidal probes adhered to MoS_2_. Top views (c) of colloidal probes adhered to graphene. The corresponding local magnification images of the colloidal probes are shown in (b) and (d).

| 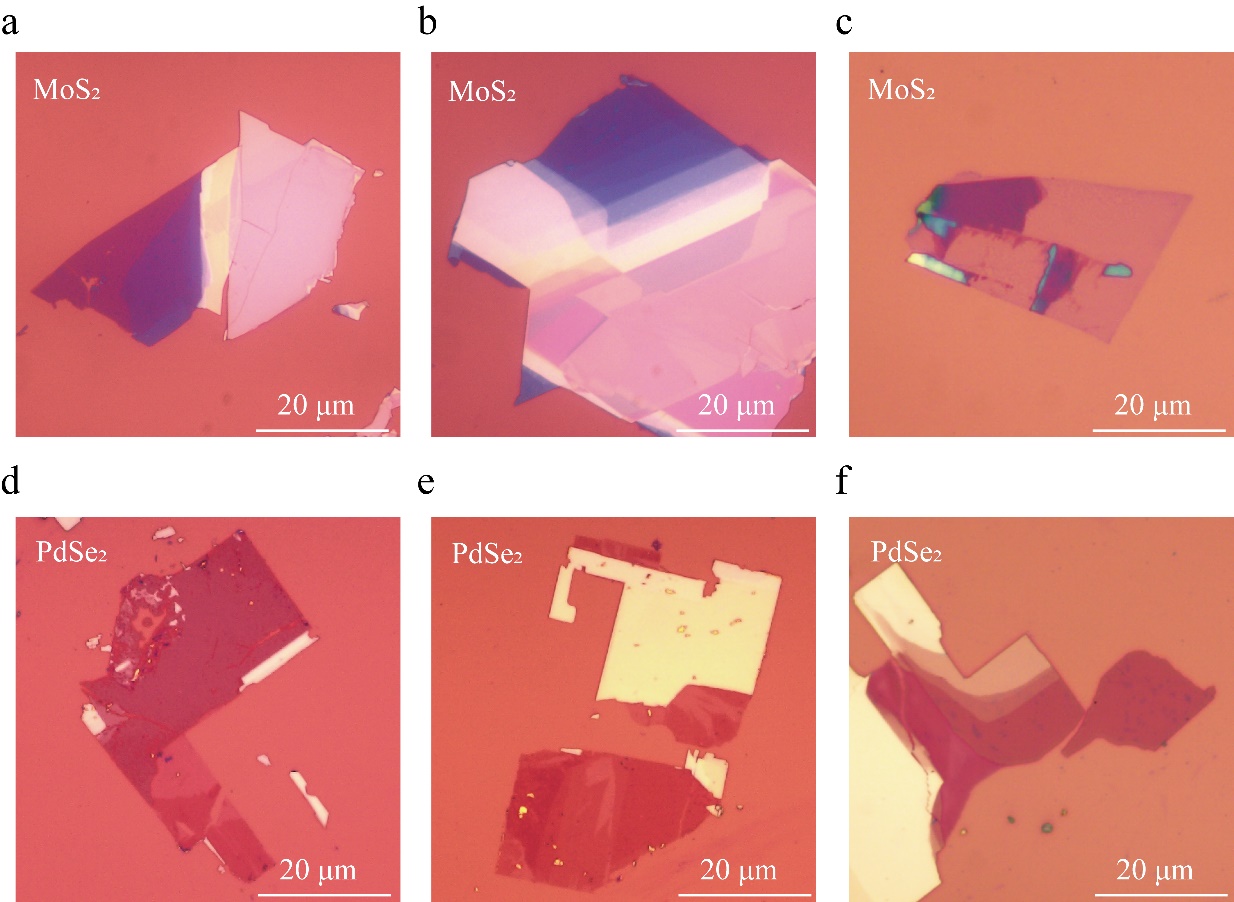 |
| --- |

Figure S3. Optical microscope images of mechanically exfoliated materials. a-c Metallographic microscopy images of few-layer 2D MoS_2_ on a Si/SiO_2_ substrate. d-f Metallographic microscopy images of few-layer 2D PdSe_2_ on a Si/SiO_2_ substrate.

| 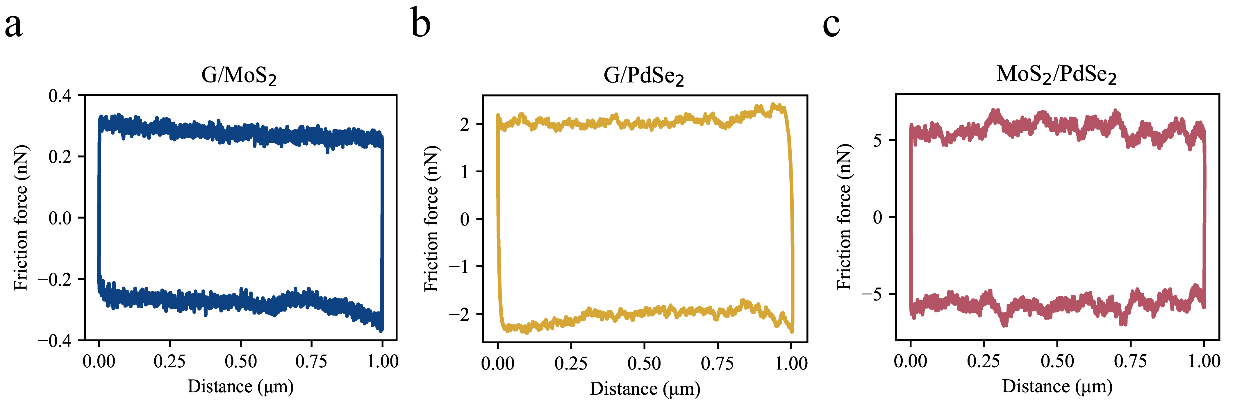 |
| --- |

Figure S4. Typical AFM friction loop images. a-c Friction loop curves of three heterogeneous systems, G/MoS_2_ (a), G/PdSe_2_ (b), and MoS_2_/PdSe_2_ (c), under a constant load of 50 nN and a scanning speed of 2 μm/s.

| 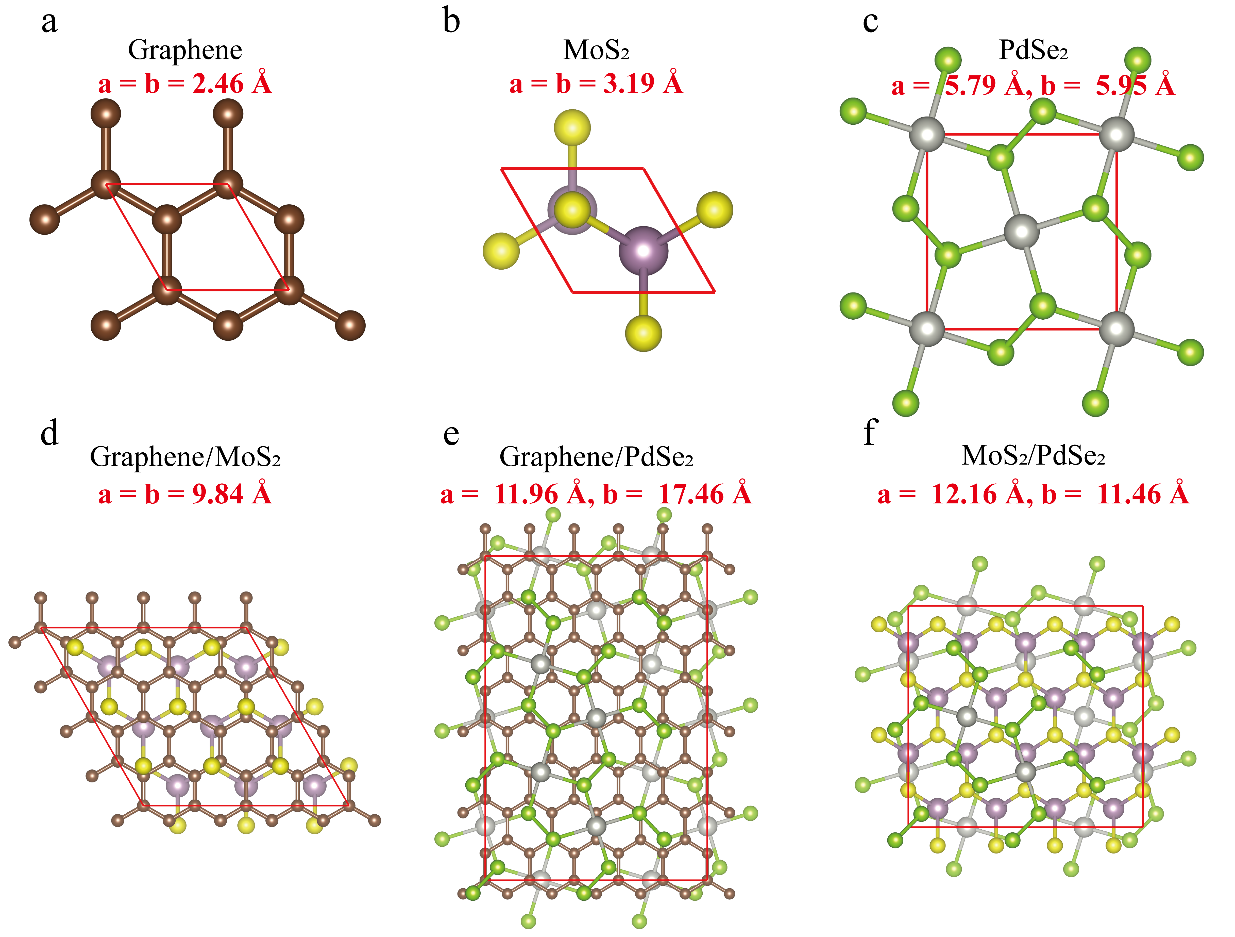 |
| --- |
| Figure S5. The schematic diagram of the structure, with the corresponding lattice constants labelled in red font. |

To further elucidate the electronic properties of the heterojunction structures, we constructed charge density difference contour plots for the three heterojunction systems, as shown in Figure S6a-c. The charge density difference in a heterojunction structure is defined as $\Delta\rho=\rho_{AB}-\rho_{A}-\rho_{B}$, where $\rho_{A}$ and $\rho_{B}$ are the charge densities of the respective single-layer structures, and $\rho_{AB}$ is the charge density of the heterostructure. It is evident that the charge density around the atoms is no longer uniform, indicating charge redistribution near the interfaces. In the case of the G/MoS_2_ heterostructure, minimal charge transfer occurs between the layers. In contrast, the MoS_2_/PdSe_2_ heterostructure exhibits substantial charge transfer between the layers across the entire interface space, suggesting significant interactions at the interface, consistent with the observed high frictional resistance in experiments^1^. In general, the direction of the built-in electric field is induced by the dipole moment. We calculated the electrostatic potential at the heterojunction interface, as shown in Figure S6d-f.

| 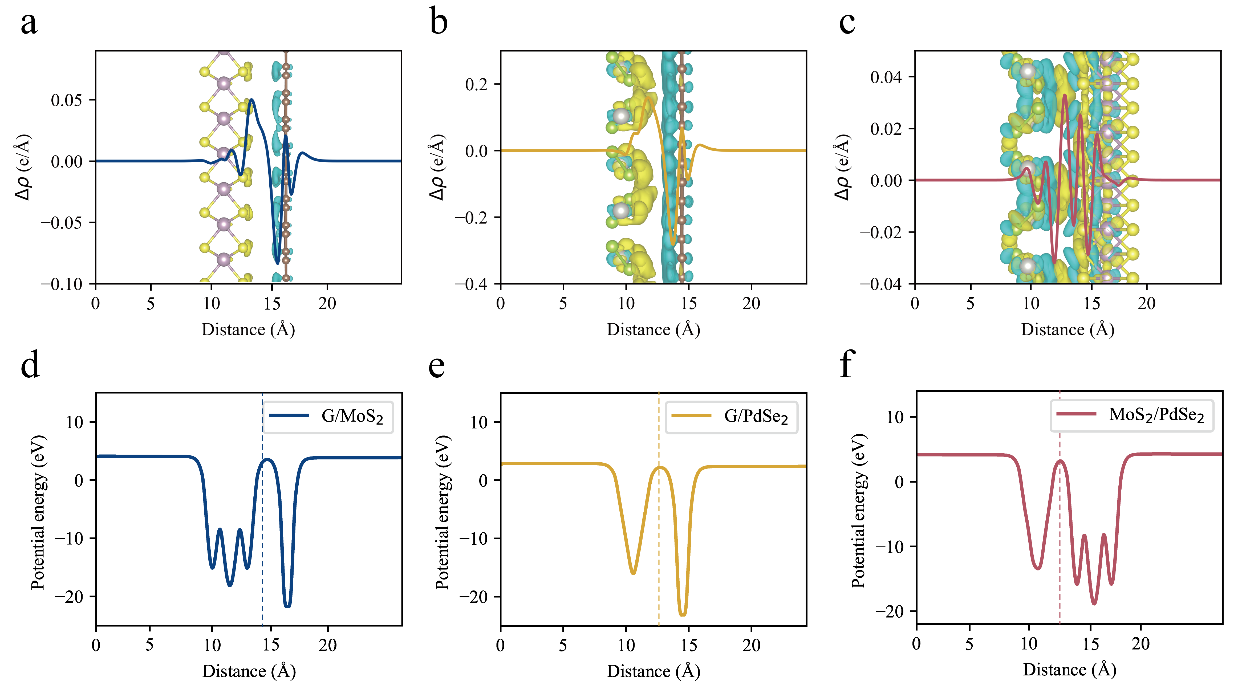 |
| --- |

Figure S6. Charge transfer and electrostatic potential at heterostructure interfaces. **a-c** The differential charge density of the heterostructure systems G/MoS_2_ (a), G/PdSe_2_ (b), and MoS_2_/PdSe_2_ (c) was obtained through DFT calculations. The solid lines in the figure represent the plane-averaged differential charge density along the z-axis direction. The blue and yellow areas represent charge consumption and accumulation, respectively. The isosurface value is 0.0002 e/Bohr^3^. **d-f** The electrostatic potential distribution along the z-axis for the G/MoS_2_ (d), G/PdSe_2_ (e), and MoS_2_/PdSe_2_ (f) heterostructure systems.

A detailed analysis of charge transfer across three distinct heterostructure systems was conducted to more accurately elucidate the phenomenon of interlayer polarization. Through quantitative calculations and visual analysis (refer to Figure S7), minimal charge transfer is observed within the G/MoS_2_ system (as shown in Figure S7a). Here, the electron gain and loss are predominantly confined to the MoS_2_ layer, aligning with the findings presented in Reference^2^. In stark contrast, the G/PdSe_2_ and MoS_2_/PdSe_2_ systems exhibit more pronounced interlayer charge transfers, characterized by the emergence of distinct positive and negative charge centers. Notably, the extent of charge transfer in the G/PdSe_2_ system is smaller, which correlates with its relatively lower interlayer friction and adhesion forces.

| 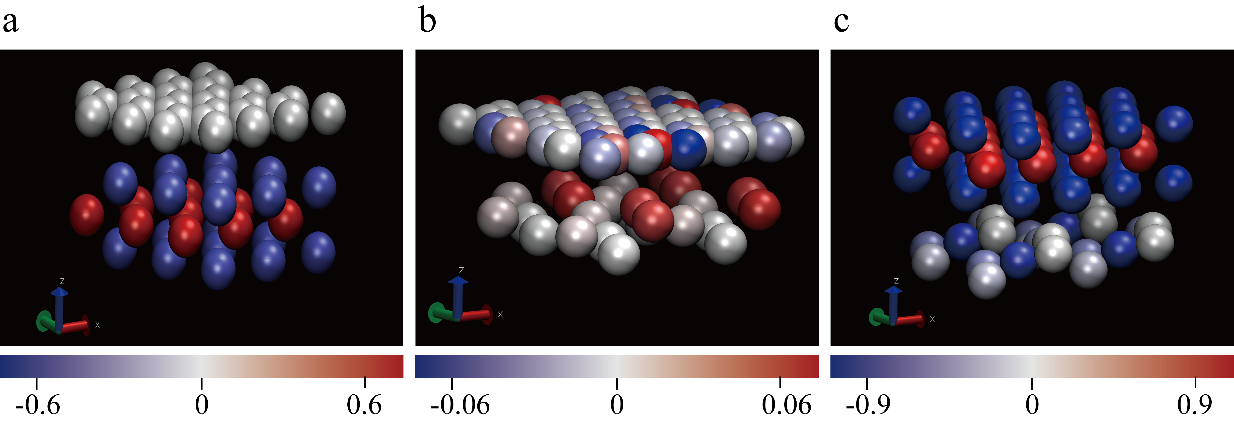 |
| --- |
| Figure S7. Visualization of Bader charge in three heterostructure systems: G/MoS_2_(a), G/PdSe_2_(b), and MoS_2_/PdSe_2_(c). Blue indicates a negative charge, suggesting that atoms gain electrons, whereas red denotes a positive charge, indicating that atoms lose electrons. |

| 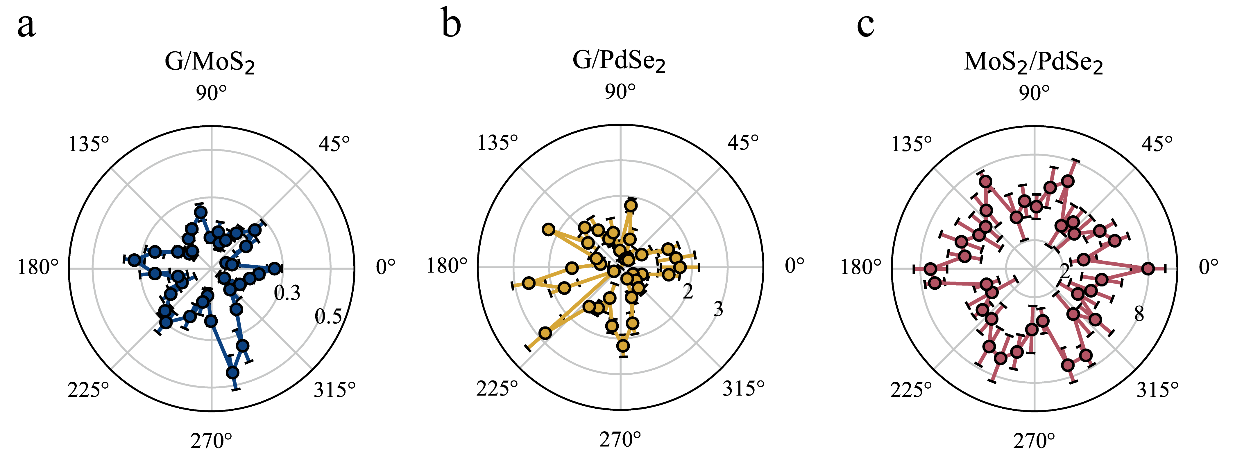 |
| --- |

Figure S8. Polar plots depicting the rotational anisotropy of friction measurements in three heterogeneous structures. AFM measurements of interlayer friction in the heterostructure systems G/MoS_2_ (a), G/PdSe_2_ (b), and MoS_2_/PdSe_2_ (c) as a function of the stacking angle.

| **** |
| --- |

Figure S9. MD simulation during the sliding process showing the variation in the slider's center of mass in the z-direction for the three systems.

The change in interlayer distance during sliding in the MoS_2_/PdSe_2_ system is significantly greater than that in the other systems, indicating a transition of the material from a higher to a lower energy state or a shift between different energy levels. This substantial variation in the interlayer distance suggests that a considerable energy barrier must be overcome during the transition from a high to a low energy state. This corresponds with the notable sliding barrier exhibited by the MoS_2_/PdSe_2_ system, as depicted in Figure 9i of the main text.

Supplementary Notes

Supplementary Note 1

The specific operational steps for preparing few-layer two-dimensional PdSe_2_ samples using the gold-assisted mechanical exfoliation method are as follows:

1. First, high-temperature-resistant double-sided tape was neatly attached to a metal-coated plate. A 6 ~ 8 cm long piece of Scotch tape was removed, the tape was stuck with the adhesive side up, and then tweezers were used to gently place a bulk PdSe_2_ sample on the Scotch tape. After being lightly pressed, the sample was removed. Thin flakes of PdSe_2_ adhere to the Scotch tape. This step was repeated until the Scotch tape was covered with thin flakes of PdSe_2_. A new piece of Scotch tape was placed on the Scotch tape with previously adhered PdSe_2_ flakes and pressed to form a bilayer structure. The double-layer Scotch tape was peeled off. This process was repeated 4-5 times, and then, heat-resistant double-sided tape was used to cover the metal-coated substrate with the repeatedly exfoliated PdSe_2_ sample.
2. The metal-coated plate with the adhered PdSe_2_ flakes was placed into a vacuum deposition machine for gold film deposition. The parameters were set to a pressure of 10^-4^ ~ 10^-5^ Pa and a deposition rate of 0.1 ~ 0.2 Å/s. This ensures uniform contact between the gold atoms and the material surface. Deposition was stopped when the gold film thickness reached approximately 200 nm. At this point, the top surface of the metal-coated substrate is covered by a gold film.
3. Heat-release tape was added to the gold film surface, pressed for a few minutes, and slowly peeled off in one direction. Due to the strong interaction forces between the $Au$ atoms and the top $Se$ atoms of PdSe_2_, when the gold film on the heat-release tape is slowly peeled off, the top layer of single-layer or few-layer two-dimensional PdSe_2_ is exfoliated.
4. The heat-release tape was placed with adhered Au/PdSe_2_ onto the target substrate (in this experiment, Si/SiO_2_ was used as the substrate). After pressing for a few minutes to ensure uniform adhesion, the silicon wafer was placed on the sample heating stage of a multifunctional high-precision two-dimensional material transfer device. After heating at 90 ℃ for approximately 5 minutes, the heat-release tape automatically detached, leaving the Au/PdSe_2_ firmly attached to the Si/SiO_2_ substrate.
5. The Si/SiO_2_ substrate was immersed with adhered Au/PdSe_2_ in an iodine-potassium iodide/deionized water etching solution for approximately 10 minutes. This removes the gold film from the surface of the ultrathin two-dimensional PdSe_2_ material, leaving few layers of PdSe_2_ on the Si/SiO_2_ substrate.
6. The Si/SiO_2_ substrate was rinsed with the adhered few-layer PdSe_2_ material in deionized water and immersed in hot acetone for approximately 30 minutes to remove residual contaminants. The tube was removed and rinsed with deionized water before being placed in isopropanol for approximately 10 minutes to remove residual acetone solution. The sample was collected and immersed in deionized water for another 10 minutes.
7. Finally, the sample was placed in a single-zone tube furnace, the temperature was set to approximately 300°C, and vacuum annealing was performed for approximately 2 hours to thoroughly remove any remaining organic residues from the sample.

Supplementary Note 2

The loss function, $L$, is constructed by combining the differences between the predicted and actual values of energies, forces, and stresses, with the following expression^3^:

| $\begin{matrix} L(z)=\lambda_{e}\left(\frac{1}{N_{str}}\sum_{n=1}^{N_{str}} \left(U^{NEP}\left(n,z\right)-U^{tar}\left(n\right)\right)^{2}\right)^{1/2} \\ +\lambda_{f}\left(\frac{1}{3N}\sum_{i=1}^{N} \left(F_{i}^{NEP}\left(z\right)-F_{i}^{tar}\right)^{2}\right)^{1/2} \\ +\lambda_{v}\left(\frac{1}{6N_{str}}\sum_{n=1}^{N_{str}} \sum_{\mu\nu} \left(W_{\mu\nu}^{NEP}\left(n,z\right)-W_{\mu\nu}^{tar}\left(n\right)\right)^{2}\right)^{1/2} \\ +\lambda_{1}\frac{1}{N_{par}}\sum_{n=1}^{N_{par}} \left\vert z_{n}\right\vert\\ +\lambda_{2}\left(\frac{1}{N_{par}}\sum_{n=1}^{N_{par}} z_{n}^{2}\right)^{1/2} \end{matrix}$ |  |
| --- | --- |

Here, $N_{\text{str}}$is the number of structures in the training data set (if a full batch is used) or the number of structures in a mini-batch, and $N$ is the total number of atoms in these structures. $U^{\text{NEP}}\left( n,z \right)$ and $W_{\mu\nu}^{\text{NEP}}\left( n,z \right)$ are the per-atom energy and virial tensor predicted by the NEP model with the parameter $(z)forthe(n^{\text{th }})$ structure, respectively, and $F_{i}^{\text{NEP }}\left( z \right)$ is the predicted force for the $i^{\text{th }}$ atom. $U^{\text{tar }}\left( n \right)$, $W_{\mu\nu}^{\text{tar }}\left( n \right)$, and $F_{i}^{\text{tar }}$ are the corresponding target values. That is, the loss terms for energies, forces, and virials are defined as the respective RMSE values between the NEP predictions and the target values. The last two terms represent the $\mathcal{L}_{1}$ and $\mathcal{L}_{2}$ regularization terms of the parameter vector. The weights $\lambda_{e}$, $\lambda_{f}, \lambda_{v}$, $\lambda_{1}$, and $\lambda_{2}$ are tunable hyperparameters.

**References**

1. Fang Q*, et al.* Interfacial electronic states and self-formed p–n junctions in hydrogenated MoS_2_/SiC heterostructure. *J Mater Chem C* **6**, 4523-4530 (2018).

2. Pierucci D*, et al.* Band Alignment and Minigaps in Monolayer MoS_2_-Graphene van der Waals Heterostructures. *Nano Lett* **16**, 4054-4061 (2016).

3. Fan, Z. et al. Neuroevolution machine learning potentials: Combining high accuracy and low cost in atomistic simulations and application to heat transport. Phys Rev B 104, 104309 (2021).
